# Assimilation of the seabird and ship drift data in the north-eastern sea of Japan into an operational ocean nowcast/forecast system

**DOI:** 10.1038/srep17672

**Published:** 2015-12-03

**Authors:** Yasumasa Miyazawa, Xinyu Guo, Sergey M. Varlamov, Toru Miyama, Ken Yoda, Katsufumi Sato, Toshiyuki Kano, Keiji Sato

**Affiliations:** 1 Application Laboratory, Japan Agency for Marine-Earth Science and Technology, Showa-machi, Kanazawa-ku, Yokohama, 236-0001, Japan; 2 Center for Marine Environmental Studies, Ehime University, Bunkyo-cho, Matsuyama, Ehime, 790-8577, Japan; 3 Graduate School of Environmental Studies, Nagoya University, Furo-cho, Chikusa-ku, Nagoya, 464-8601, Japan; 4 Atmosphere and Ocean Research Institute, the University of Tokyo, Kashiwa, Chiba, 277-8564, Japan; 5 National Maritime Research Institute, Shinkawa, Mitaka, Tokyo, 181-0004, Japan

## Abstract

At the present time, ocean current is being operationally monitored mainly by combined use of numerical ocean nowcast/forecast models and satellite remote sensing data. Improvement in the accuracy of the ocean current nowcast/forecast requires additional measurements with higher spatial and temporal resolution as expected from the current observation network. Here we show feasibility of assimilating high-resolution seabird and ship drift data into an operational ocean forecast system. Data assimilation of geostrophic current contained in the observed drift leads to refinement in the gyre mode events of the Tsugaru warm current in the north-eastern sea of Japan represented by the model. Fitting the observed drift to the model depends on ability of the drift representing geostrophic current compared to that representing directly wind driven components. A preferable horizontal scale of 50 km indicated for the seabird drift data assimilation implies their capability of capturing eddies with smaller horizontal scale than the minimum scale of 100 km resolved by the satellite altimetry. The present study actually demonstrates that transdisciplinary approaches combining bio-/ship- logging and numerical modeling could be effective for enhancement in monitoring the ocean current.

Development of Global Positioning Systems (GPS) allows us to easily recognize accurate positions of objects moving over the Earth through GPS loggers. GPS is now frequently used for tracking animals in outdoor field (bio-logging). Analysis of GPS log data and/or combined use of GPS with various kinds of sensors in animal tracking further allow measurements of oceanic conditions by bio-logging^1^. Recently a Japanese biologist group has succeeded to reproduce ocean current structure in the north-eastern sea of Japan (off Tohoku and Hokkaido regions; Fig. 1a,b) by analyzing drift data calculated from GPS logs of a species of seabird, the streaked shearwater, *Calonectris leucomelas*^2^. During their foraging trips, the seabirds spent half of their time resting on the sea surface and tended to be passive drifters^2^. They found that the seabird drift actually represented the gyre mode Tsugaru warm current seasonally developing from summer to autumn seasons^3^. This study investigates feasibility of the seabird drift assimilation into an ocean general circulation model. Traditional surface drifting buoy data obtained in the same periods (Fig. 1c,d) are utilized for validation.

In addition to the seabird drift, we assimilate the ship drift data (Fig. 1e) into the same ocean model targeting the same region and the same phenomenon, and compare its impact on the model skill with that of the seabird drift. The ship drift, which is calculated from difference between the ship speed over ground and that through water, has been traditionally used for observing the ocean current distribution^4^. A Japanese marine technologist group has recently developed a shipping support system for coastal vessels utilizing environmental data including ocean current forecast for effective ship operation^5^. They have also developed a monitoring system of the ship drift (ship-logging) around the Japan coastal ocean^6^. We examine feasibility of skill improvement in the ocean current product by assimilating the ship drift data into the model.

An operational ocean nowcast/forecast system, Japan Coastal Ocean Predictability Experiment 2 (JCOPE2^7^; see http://www.jamstec.go.jp/jcope/for real-time forecast), whose products have been widely used by oceanographic scientists^8^ and engineers^9^ is adopted for investigating impacts of seabird and ship drift assimilation on the model skill. A recently developed assimilation scheme of the surface velocity^10^ is applied to the drift data in this study. The JCOPE2 system basically assimilates a huge number of observation data: satellite sea surface height anomaly, satellite sea surface temperature, and in-situ temperature and salinity profiles^7^. This study aims at investigating additional impacts of the drift data on the model skill.

There have been several studies on the drifter data assimilation. Various methods including optimal interpolation^11^, nudging^12^, Kalman filter^13^,^14^, variational methods^15^,^16^ show all skillful results. The assimilation methods also have two types of the observation data forms to be used: Lagrangian trajectory^15^ or diagnosed Eulerian velocity^16^. Our method is variational one based on inclusion of Eulerian velocity supposing geostrophic current approximation (see methods).

## Results

### Impacts of the seabird and ship drift data assimilation

Figure 2a compares temporal mean surface current distribution at 5 m depth without (left panel) and with (right panel) assimilation of the seabird drift data in the 2010 season. Even without the assimilation of the seabird drift, both the model current and seabird drift exhibit good agreement with each other. In particular, southwestward current forming a southeastern part of the clockwise eddy around 40.5° N, 142.5° E, which is associated with the gyre mode Tsugaru warm current^3^, and southward current around 39.2° N, 142.2° E are quite similar to each other. Even without the assimilation of the seabird drift data, such kind of comparatively large scale southwestward and southward currents are generally represented well by the assimilation of the usual observation data^7^,^8^.

The gyre mode Tsugaru warm current is characterized as a light current exiting from an eastward oriented channel perpendicular to the coastline^17^. The gyre size is approximated as 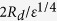, where 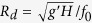, 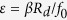; 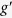 is reduced gravity; 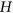 is downstream thickness of light layer; 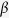 is latitudinal variation of the Coriolis parameter 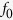. Under the condition with sufficiently large 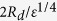, the gyre mode appears. A typical 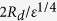 value estimated for the Tsugaru warm current during the warm months is 110 km^17^, which is close to the scales of the clockwise eddies shown in Fig. 2. We find that the assimilation of SSHA working to correct the subsurface stratification related with 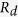 (see methods) is basically important for representation of the gyre mode in our nowcast/forecast system.

The seabird drift assimilation enhances eastward current around 41.5° N, 142.5° E and southward current around 41.5° N, 143° E (Fig. 2a). Such impact of the assimilation is clearly depicted in Fig. 3a (also in left panel of Fig. 2a) showing difference in mean surface current during the target period. Figure 3a indicates that the clockwise eddy and adjacent two counter-clockwise eddies north and south of the clockwise eddy are generally intensified by the additional input of the seabird drift information. Model skills represented by correlation and root mean square error (RMSE) to the seabird drift are reasonably improved by the assimilation, and in particular, the skill for non-assimilated drifting buoy data is notably improved (Table 1).

The growth of the gyre mode is also seen in the 2011 season (Fig. 2b). The assimilation of the seabird drift (right panel of Fig. 2b) seems to affect the model result less than that in the 2010 season (right panel of Fig. 2a). Representation of the clockwise eddy in the drift data assimilation result is modified by weakening eastward current around the northern edge and by strengthening westward current around the southern edge as indicated in Fig. 3b and left panel of Fig. 2b. Model skill for the seabird drift is improved by the assimilation; however, the skill for non-assimilated drifting buoy data is not good, and not much improved by the assimilation (Table 2).

Assimilation of the ship drift data causes eastward extension of the clockwise eddy shape (Fig. 2c). Impact of the assimilation is represented by intensification of both the clockwise eddy and an adjacent counter-clockwise eddy (Fig. 3c and left panel of Fig. 2c). Skill improvement in the ship drift assimilation is caused only by the drift component along navigation direction not by that normal to navigation direction (Table 3). The wind-driven lateral drift of the ship^4^ that could be included in the drift component normal to navigation direction may induce worse skill compared to the skill for the drift along navigation direction.

### Relation of the drift to the wind

Both of the seabird and ship drift data could be affected by the wind-induced slip caused by wind blowing directly on a surface body, the wave-induced Stokes drift, and the wave-breaking effect as investigated for the surface drifting buoys^18^. Estimates of correlation between the seabird drift and the wind indicate that positive correlation is statistically significant for the data in 2010 and 2011 and the correlation value in 2011 is higher than that in 2010 (Table 4), suggesting that the wind effects are not negligible for the seabird drift. The maximum wind magnitude in the 2011 season is larger (exceeding 15 m s^−1^) than that in the 2010 season because of a typhoon staying around the target region in the beginning of September 2011 (not shown). The severe atmospheric disturbances could affect contribution of the wind effects to the seabird drifting. The behavior of the drifting buoys might be also affected partly by the severe atmospheric disturbances^18^ as indicated by a small but significant positive correlation value in the 2011 season. The ship drift in the 2014 season shows no significant correlation with wind (Table 4), but estimates from a longer term period indicate positive and significant correlation values, especially for the drift component normal to navigation direction.

Magnitude of the wind-induced slip is proportional to a ratio (Aa/Aw) of the areas above (Aa) and below (Aw) the sea surface: Aa/Aw < 1/40 for the drogued buoys^18^, Aa/Aw~1/3 for the seabird^2^, and Aa/Aw ranges from 4 to 19 depending on the wind direction relative to the navigation direction for a ferry boat^4^. The ship drift is thus basically affected by the wind-induced slip due to the relatively larger area of the body above the surface, and the seabird drift is affected more by the wind-induced slip especially during the strong wind period than the drogued buoys. To further check this issue, we compare the modified seabird drift by subtracting regressed values from the wind to the drift for the 2010 (regression coefficient from the wind to the drift is 0.007; Table 1) and 2011 seasons (regression coefficient is 0.009; Table 2). The skill in the 2011 season is improved while that in the 2010 season becomes worse slightly, inferring that the wind effects are more dominant in the 2011 season as compared to in the 2010 season. This is consistent with the analysis by Yoda *et al.*^2^ showing that the frequency distribution of the seabird drift data is more similar to that of the ocean current data obtained by ADCP in the 2010 season as compared to the case in the 2011 season.

To examine possible effects from the wind-induced slip on the ship drift, we assimilate the ship drift along navigation direction alone. The skills become improved especially for the drift along navigation direction (Table 3), and are generally better than those in the assimilation of all the components of the ship drift. By assimilating the drift along navigation direction alone, we are able to effectively exclude the effects from the wind driven lateral drift^4^.

### Sensitivity experiments

Fitting of the seabird drift obtained in the 2010 season to the model is comparatively better than that in the 2011 season and the ship drift in the 2014 season (Tables 1,2, and 3), indicating that in the 2010 case the geostrophic current component included in the drift is effectively assimilated into the model. Since the fitting of the ‘independent’ buoy drift data to the model is also good in the 2010 case (Table 1), the buoy drift data could be utilized for checking performance of the assimilation parameters (see methods). We test several choices of the parameters: 0.02, 0.1 (default), 0.4 of the drift observation error (in m s^−1^), and 25, 50 (default), 100 of the horizontal scale (in km) included in the background error covariance.

Higher correlation and lower RMSE are obtained with smaller observation error setting (supplementary Fig. 1a); however, the small observation error of 0.02 m s^−1^ leads to worse fitting to the other observation data including SSHA, SST, in-situ temperature and salinity (supplementary Fig. 1b). Overfitting to the drift data distorts the density structure of the model by assimilating ageostrophic and/or wind/wave-induced components contained in the drift as misinterpreted geostrophic component.

Though the smaller horizontal scale improves the fitting to the all observation data (supplementary Fig. 2a), the choice of a 50 km scale indicates the smallest RMSE and a smaller 25 km scale does not improve the skill (supplementary Fig. 2b). This means that the seabird drift is capable of conveying the information on the minimum 50 km scale phenomena to the model. Figure 2a,b indicate that the flow structures with horizontal scale smaller than that of the clockwise eddy (110 km) associated with the gyre mode are actually modified by the assimilation of the seabird data. Note that the minimum horizontal scale of 50 km is smaller than that represented by SSHA data, 100 km^19^, and the hourly interval of the drift data is smaller than the typical temporal interval of SSHA data, 10 days^19^. The seabird and ship drift sampling points existing within gaps of the satellite altimetry tracks shown in Fig. 4 further suggest possibility of the drift observation enhancing the observation network for capturing the mesoscale phenomena that has been maintained by the satellite altimetry so far.

## Discussion

The data assimilation scheme designed for extracting information on the geostrophic current component from the seabird and ship drift data works well with the proper choice of assimilation parameters, and modifies intensity and shape of the clockwise eddy. The seabird drift assimilation in the 2010 season well fits the seabird drift and leads to the considerable skill improvement in comparison with the non-assimilated buoy drift. In contrast, fitting of the seabird drift in the 2011 season and the ship drift in the 2014 season is worse than the case in 2010. This is partly because the seabird drift in the 2011 season is affected more by the wind/wave-induced effects than that in the 2010 season with the comparatively moderated wind condition, and the ship drift is more sensitive to the wind/wave-induced effects due to a larger ratio of the area above and below the sea surface (Aa/Aw) as compared to those of the seabird and buoy drifts.

The preliminary analysis of this study suggests possible wind/wave induced effects on the drift depending on Aa/Aw ratio of the drifting body and the atmospheric condition. Future works include more careful consideration of the wind/wave induced effects possibly included in the seabird and ship drift data. Evaluation of the wind-induced ship drift^4^, and field observation of the seabirds focusing on the wind-induced slip and/or other wind/wave induced effects could be planned for more effective utilization of the drift data.

The present study focuses on the assimilation of the drift data in the relatively deep ocean (with bottom depth deeper than 800 m). The drift in the shallow region are thus excluded from the data assimilation scheme. The definition of the assimilation scheme (1) assumes that the present assimilation utilizes only the geostrophic component contained in the drift data, which possibly includes the ageostrophic components. More sophisticated data assimilation methods such as Ensemble Kalman Filter^14^ and/or 4DVAR^16^ allowing the assimilation of the drift data in more comprehensive manner will be applied to the drift data assimilation in the future.

If we establish dynamically combined social systems between the seabird and ship drift monitoring and the operational ocean nowcast/forecast suggested by the present study, the systems could involve a positive feedback mechanism toward enhancing monitoring network. The ocean nowcast/forecast information with improved skill by assimilating the drift data would be helpful for studying behaviors of marine animals^1^ and effective ship navigation using it, hopefully resulting in higher quality wildlife management and more reduced use of fuel in ships together with more reduce emission of CO_2_^5^. Monitoring people have an incentive to extend the monitoring coverage and increase the density for receiving more improved nowcast/forecast products. We emphasize a possible role of the operational ocean nowcast/forecast as a medium in developing a transdisciplinary sustainable ocean monitoring network.

## Methods

### Ocean forecast system JCOPE2

The targeted ocean nowcast/forecast system, JCOPE2, is based on an ocean general circulation model with generalized sigma coordinate in vertical^20^, and covers the Western North Pacific (10.5°−62° N, 108°−180° E) with horizontal resolution of 1/12 deg. and 46 vertical levels from surface to maximum 6500 m depth. To represent realistic oceanic current and eddy states as observed, JCOPE2 assimilates satellite sea surface height anomaly (SSHA), satellite sea surface temperature, and in-situ temperature and salinity into the model using a three-dimensional variational (3DVAR) method. 3DVAR minimizes a cost function composed of terms describing square distance between modeled and observed variables by adjusting control parameters: the amplitudes of temperature-salinity combined empirical orthogonal functions^21^. Further detail of the JCOPE2 system is described in the reference paper^7^.

### Drift data assimilation method

**Figure 1:**
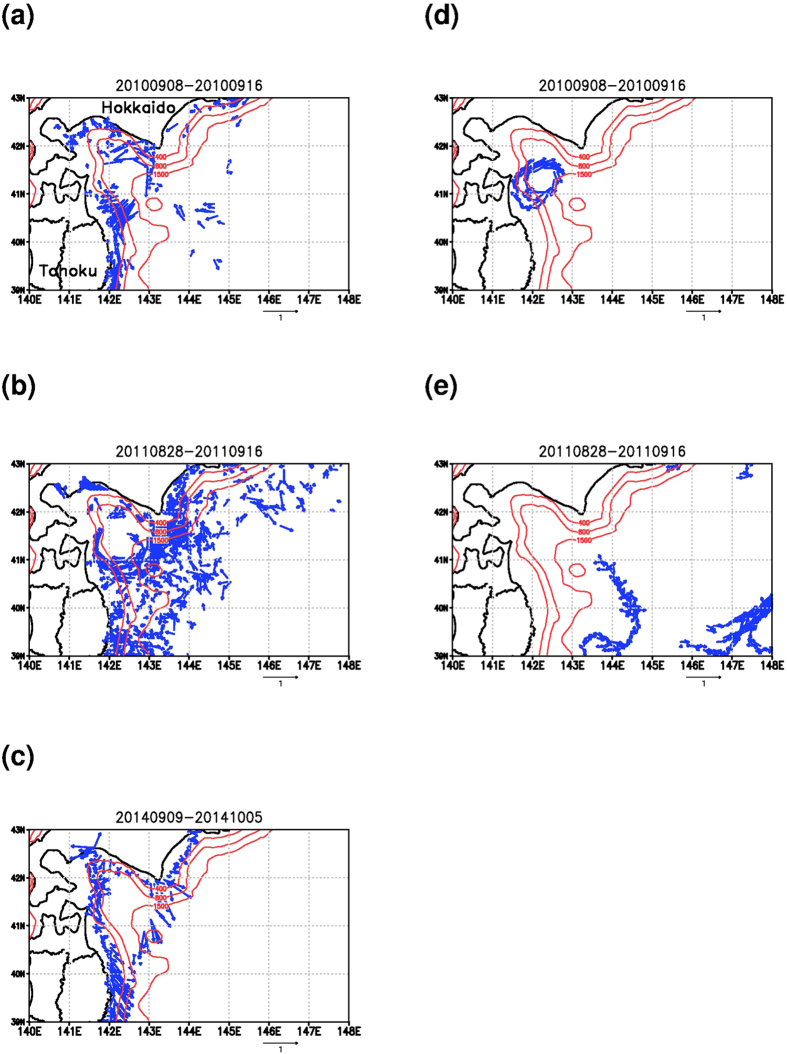
Composite maps of the seabird drift obtained in the 2010 season (a) and in the 2011 season (b), the buoy drift in the 2010 season (c) and in the 2011 season (d) and the ship drift in the 2014 season (e). Numerics at the top of the figure denote the period. Contours with red color indicate iso-depth lines with 400 m, 800 m, and 1500 m levels. These figures were created by using the Grid Analysis and Display System (GrADS) Version 2.0.

**Figure 2:**
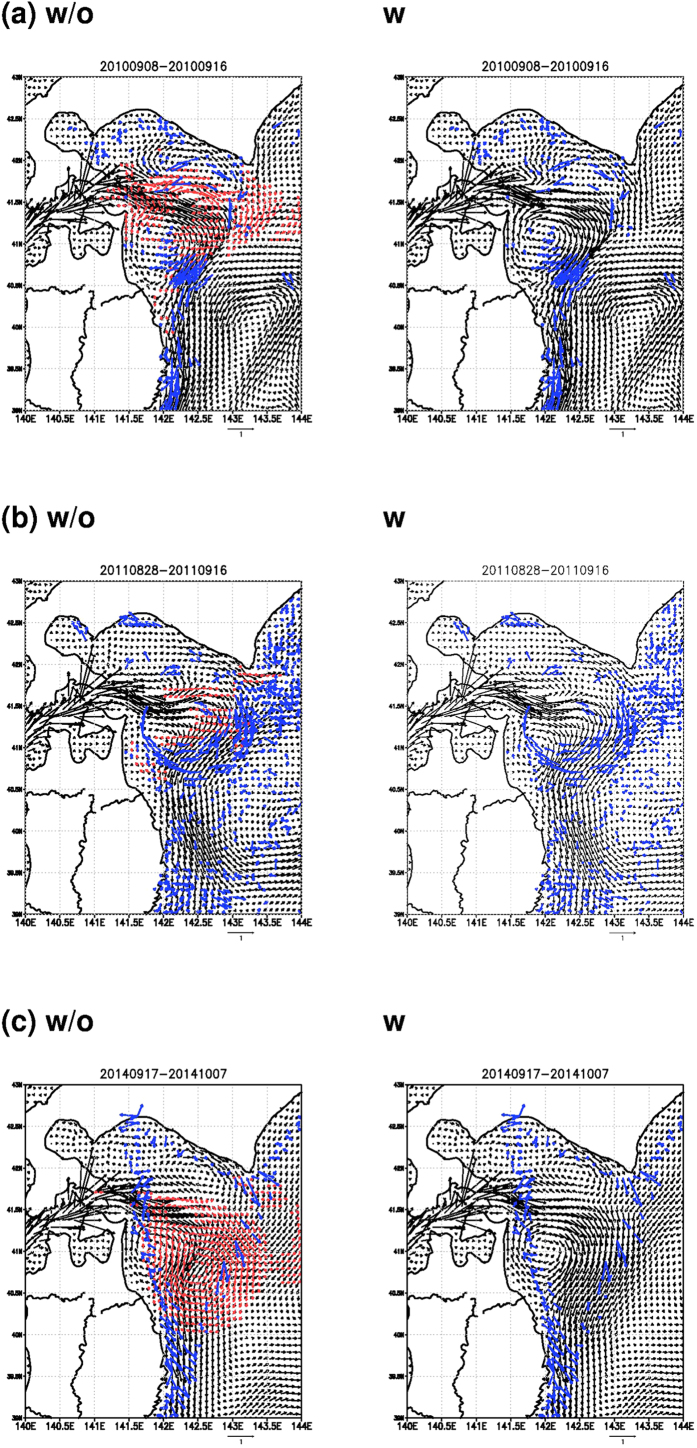
Model current distributions at 5m depth averaged during the target period. Left (right) panels indicate the results without (with) the assimilation of the seabird drift in the 2010 season (**a**), the seabird drift in the 2011 season (**b**), and the ship drift in the 2014 season (**c**). The composite drift data are represented by the blue colored vectors. Difference larger than 0.1 m s^−1^ in magnitude of the flows with and without the assimilation is indicated by the red colored vectors in the left panels. Numerics at the top of the figure denote the period. These figures were created by using the Grid Analysis and Display System (GrADS) Version 2.0.

**Figure 3:**
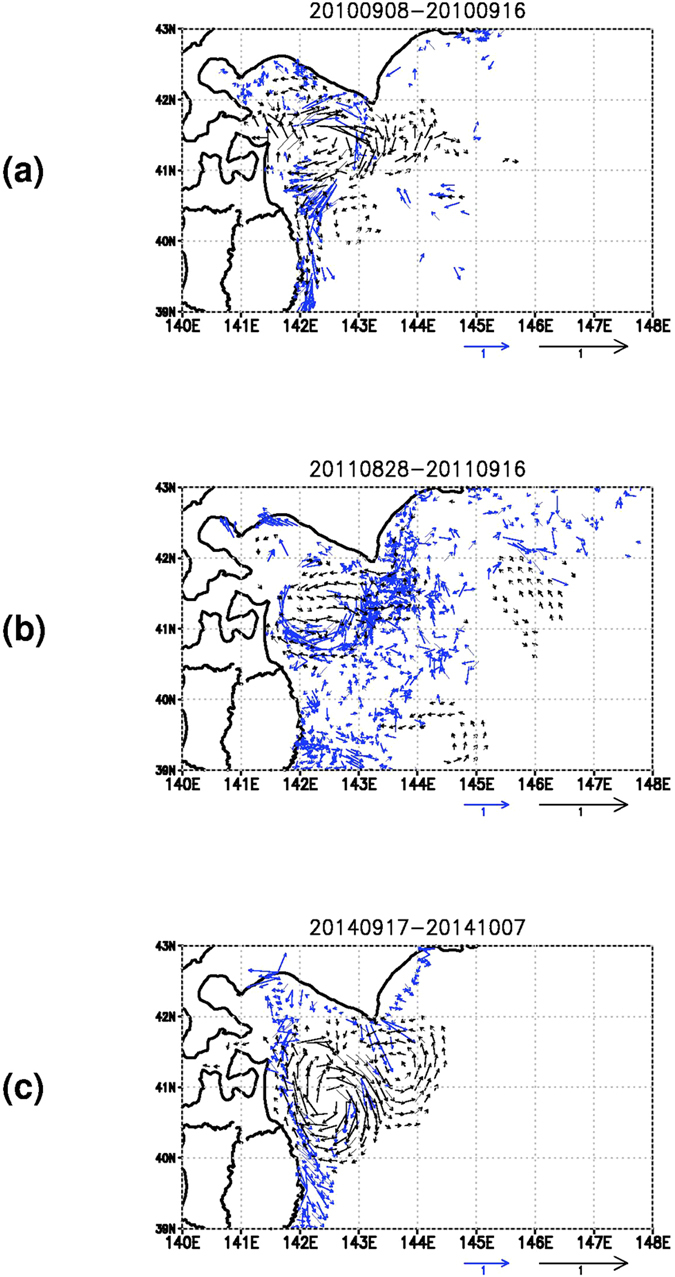
Difference between the time mean surface current at 5m depth with and without the assimilation of the seabird drift in the 2010 season (a), the seabird drift in the 2011 season, and the ship drift in the 2014 season (c). Difference with magnitude smaller than 0.05 m s^−1^ is not shown. The composite drift data are represented by the blue colored vectors. The arrow length scale for the difference is doubled as compared to that for the drift. Numerics at the top of the figure denote the period. These figures were created by using the Grid Analysis and Display System (GrADS) Version 2.0.

**Figure 4:**
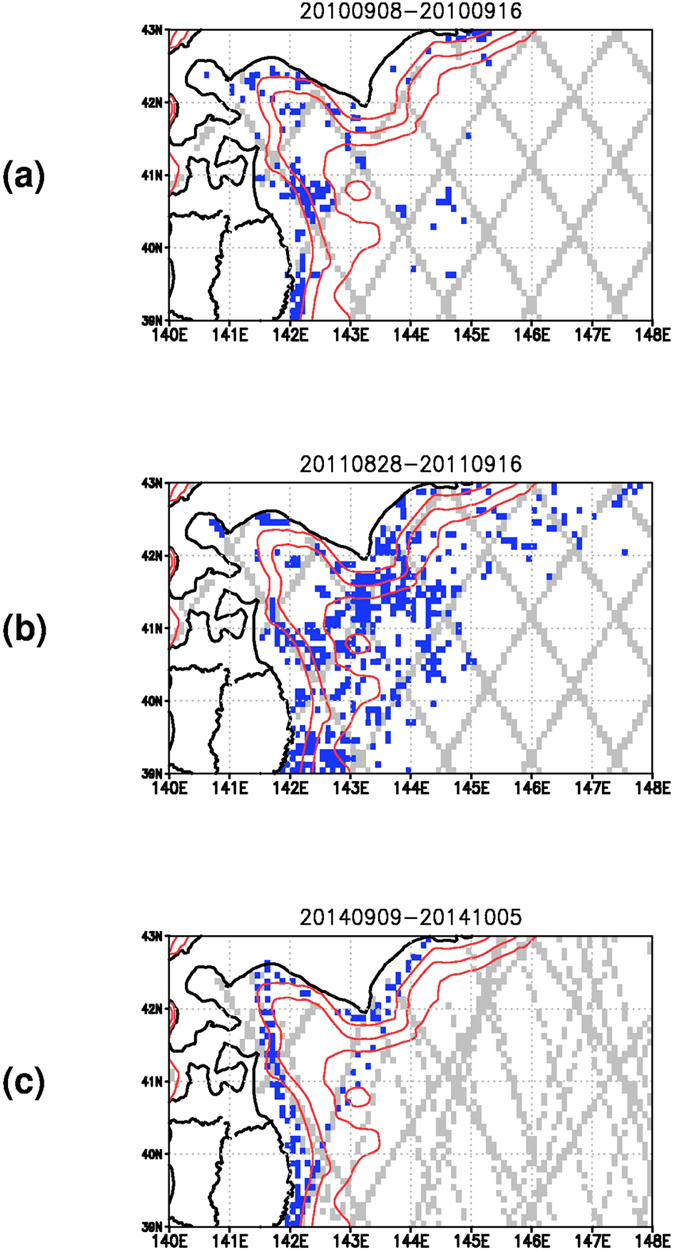
Measurement positions of the drift indicated by the blue colored dots and the satellite altimetry indicated by the gray colored dots. (**a**) The seabird drift and satellite altimetry in the 2010 season, (**b**) in the 2011 season, and (**c**) the ship drift and satellite altimetry in the 2011 season. Contours with red color indicate iso-depth lines with 400 m, 800 m, and 1500 m levels. Numerics at the top of the figure denote the period. These figures were created by using the Grid Analysis and Display System (GrADS) Version 2.0.

We add an additional term to the original cost function (see eq. (1) in the reference paper^7^):


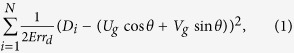


where 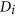 and 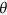 denote magnitude and direction of the drift data *i*, respectively; the direction of the drift is defined as an angle between the drift and the eastward direction. Minimization of the cost function (1) penalizes the square distance between the drift and the geostrophic current component along the drift direction. An observation error of the drift (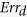) is an important parameter affecting a relative weight of the term (1) among all terms in the cost function. 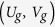 are surface geostrophic current of the model in eastward and northward direction, respectively, and they are functions of sea surface dynamic height (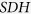):


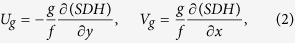


where 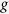 and 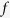 denote gravity acceleration and Coriolis parameter, respectively. 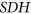 is defined referring to 800 m depth, and thus the drift obtained in regions with bottom depth shallower than 800 m (see iso-depth contours in Fig. 1) are not assimilated into the model. The choice of 800 m depth is due to a typical vertical scale of the mesoscale eddy/current structures in the target region. The additional cost function (1) was previously introduced into the JCOPE2 system for investigating feasibility of the surface drifting buoy data assimilation^10^. Note that minimization of the cost function (1) results in modifying the temperature and salinity distributions from sea surface to 800 m depth through changes in SDH based on the geostrophic current approximation (2). Minimization of the whole cost function including (1) is performed with 7 days interval^7^ assuming that the time window of the drift data is prior to and after 3 days performing it.

### Seabird drift data

Seabird drift data were calculated from the GPS logger data with 12 samples in 2010 and 24 samples in 2011 obtained by the field experiments conducted by Yoda *et al.*^2^ using the same method as reported in Yoda *et al.*^2^. Composite views of the hourly mean drift data gridded on 1/12 deg. grid are shown in Fig. 1a (the 2010 season) and 1b (the 2011 season). Clockwise circulation patterns around 41.5° N, 142.5° E shown in Fig. 1a,b represent the growth of the gyre mode Tsugaru warm current^2^. The data within one day and a 1/12 deg. grid are compared with the daily mean of the geostrophic current at the same day and the same grid in the cost function (1).

### Surface drifting buoy data

Track data of surface drifting buoys with drogues archived in Canadian Marine Environmental Data Service (MEDS) were utilized for validation of the analysis. Since the MEDS archive provides the annually compiled data set from 1979 to 2012, we used the data in 2010 and in 2011 for comparison with the seabird drift data and their associated model products. We calculated hourly velocity from the type ‘P&S’ data including drifter positions with approximately hourly interval. Composite of the buoy data during the same period in 2010 (Fig. 1c) as that of the seabird drift (Fig. 1a) indicates both the data capture a same clockwise eddy, suggesting the seabird’s ability to measure the surface current as the traditional surface drifting buoys. Comparison of the composite views during the 2011 season (Fig. 1b,d) shows no clear correspondence between each other because of almost no overlapping of the measurement coverages between them.

### Ship drift data

Ship drift data every 10 minutes were obtained from 12 cargo ships of coastal service companies by subtracting the ship speed through water measured by the ship speed meter from the ship speed over ground calculated using the GPS log and correcting systematic errors involved in the speed meter^6^. Hourly mean data were obtained within a target region (38°−44° N, 140°−149° E) during a period from 27 December 2013 to 30 October 2014. Figure 1e, visualizing a composite of the ship drift data gridded on 1/12 deg. grid during a target period from 9 September to 5 October 2014, depicts a clockwise circulation around 41.5° N, 142.5° E as shown by the seabird drift in the different years (Fig. 1a,b).

### Wind data

In addition to the JCOPE2 current data, hourly wind data 10 m above the surface provided from Japan Meteorological Agency non-hydrostatic Meso Scale Model with horizontal 5 km grid (JMA MSM^22^) were compared with the drift data for examining possible relations of the drift to the wind.

### Data assimilation experiments

We conducted data assimilation experiments targeting three periods: from 8 to 16 September 2010 (t10) and from 28 August to 16 September 2011 (t11) for the seabird drift, and from 17 September to 7 October 2014 (t14) for the ship drift. The initial conditions of the assimilation experiments were taken from the reanalysis data^7^,^8^ produced by the JCOPE2 system, and the usual observation data including the satellite sea surface height anomaly, satellite sea surface temperature, and the in-situ temperature and salinity profiles were also assimilated into the model. The gyre mode events of the Tsugaru warm water in the three periods are actually reported in Quick Bulletin Ocean Conditions provided by the Hydrographic and Oceanographic Department of the Japan Coastal Guard (http://www1.kaiho.mlit.go.jp/KANKYO/KAIYO/qboc/backnumber.html). Base experiments with the seabird drift data were performed with default 3DVAR parameters as follows: observation error of the drift data (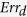
in (1)) is 0.1 m s^−1^ and horizontal scale of background error covariance (see (3) in the reference paper^7^) is 50 km for the target region (38°−44° N, 140°−149° E). Assimilation of the ship drift data for a base experiment was conducted with the observation error of 0.02 m s^−1^ and the horizontal scale of 50 km. The smaller observation error than that in the seabird drift data assimilation is chosen because it effectively improves the model skill without a counter effect on the fitting to the other types data (not shown), which occurs in the assimilation of the seabird data with the error value of 0.02 m s^−1^ (see supplementary Fig. 1 and the relevant description).

## Additional Information

**How to cite this article**: Miyazawa, Y. *et al.* Assimilation of the seabird and ship drift data in the north-eastern sea of Japan into an operational ocean nowcast/forecast system. *Sci. Rep.* **5**, 17672; doi: 10.1038/srep17672 (2015).

## Supplementary Material

Supplementary Information

## Figures and Tables

**Table 1:** Correlation values between the model current at 5m depth and the drift data for the 2010 season.

	w/o t10	w t10
seabird drift (N = 976)	**0.631** (0.309)	**0.677** (0.292)
buoy drift (N = 124)	**0.417** (0.466)	**0.655** (0.381)
seabird drift modified by regressed value from wind (N = 976)	**0.625** (0.310)	**0.672** (0.293)

‘w/o’ (‘w’) means the data assimilation cases without (with) the seabird drift. Bold font denotes statistically significant correlation with a two-tailed P value < 0.05. Numerics in parentheses indicate RMSE in m s^−1^. ‘N’ indicates a number of each data.

**Table 2:** As in Table 1 except for the 2011 season.

	w/o t11	w t11
seabird drift (N = 3674)	**0.269** (0.309)	**0.288** (0.303)
buoy drift (N = 3240)	−0.098 (0.395)	−0.096 (0.392)
seabird drift modified by regressed value from wind (N = 3674)	**0.272** (0.306)	**0.297** (0.300)

**Table 3:** As in Table 1 except for the assimilation of the ship drift in the 2014 season.

	w/o t14	w t14	with ship drift along navigation direction t14
ship drift (N = 460)	**0.295** (0.438)	**0.349** (0.427)	**0.355** (0.429)
ship drift along navigation direction (N = 230)	**0.419** (0.455)	**0.504** (0.431)	**0.517** (0.428)
ship drift along normal direction to navigation direction (N = 230)	0.056 (0.420)	0.058 (0.424)	0.065 (0.430)

**Table 4:** Correlation values between the drift data and JMA-MSM wind product.

seabird drift t10 (N = 1484)	**0.065**
seabird drift t11 (N = 4382)	**0.136**
buoy drift t10 (N = 156)	0.047
buoy drift t11 (N = 3256)	**0.066**
ship drift t14 (N = 468)	0.086
ship drift along navigation direction t14 (N = 384)	0.091
ship drift along normal direction to navigation direction t14 (N = 234)	0.115
ship drift, 2013.12.-2014.10 (N = 4514)	**0.048**
ship drift along navigation direction, 2013.12-2014.10 (N = 2257)	−0.010
ship drift along normal direction to navigation direction, 2013.12-2014.10 (N = 2257)	**0.125**

Bold font denotes statistically significant correlation with a two-tailed P value < 0.05. ‘N’ indicates a number of each data.
