# Supplementary material for: Assimilation of the seabird and ship drift data in the north-eastern sea of Japan into an operational ocean nowcast/forecast system
Source: Sci Rep. 2015 Dec 3;5:17672. doi: 10.1038/srep17672 (PMC4668391; doi:10.1038/srep17672)
Supplement: Supplementary Information [file srep17672-s1.pdf]

Title: Assimilation of the seabird and ship drift data in the north-eastern sea of Japan into an operational ocean nowcast/forecast system

Authors: Yasumasa Miyazawa, Xinyu Guo, Sergey M. Varlamov, Toru Miyama, Ken Yoda, Katsufumi Sato, Toshiyuki Kano, Keiji Sato

Supplementary Figure 1. (a) Dependence of the correlation and RMSE (in $\mathrm{m\,s^{-1}}$) of the model for the buoy drift in the 2010 season on the choice of the observation error of the seabird drift. (b) As in (a) except for dependence of the cost function terms for each type of observation data normalized by those without the drift data assimilation. These figures were created by using GNUPLOT Version 4.2.

Supplementary Figure 2. As in supplementary Fig.1 except for dependence on the choice of the horizontal scale included in the background error covariance. These figures were created by using GNUPLOT Version 4.2.

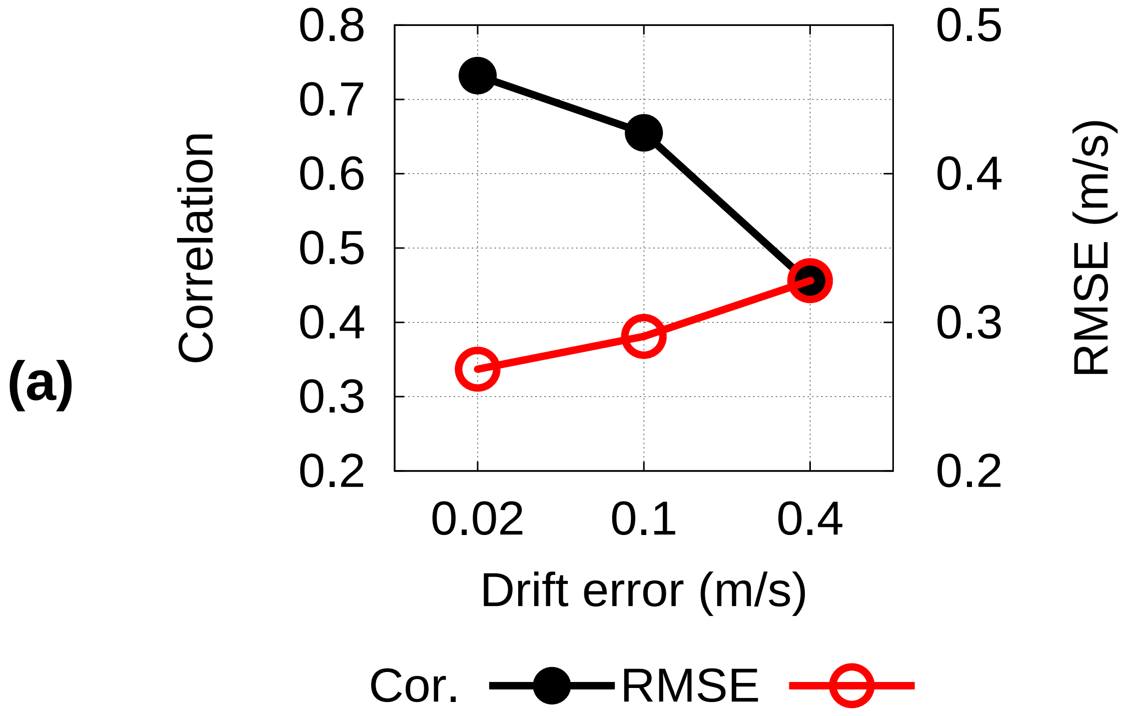

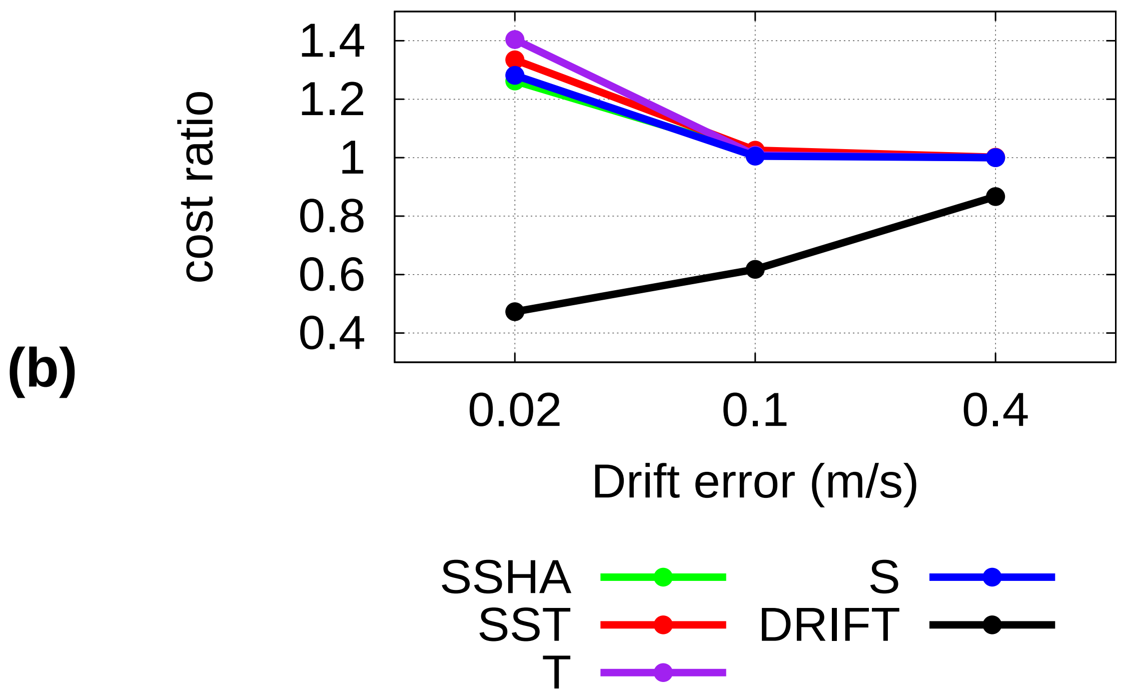

Supplementary Figure 1

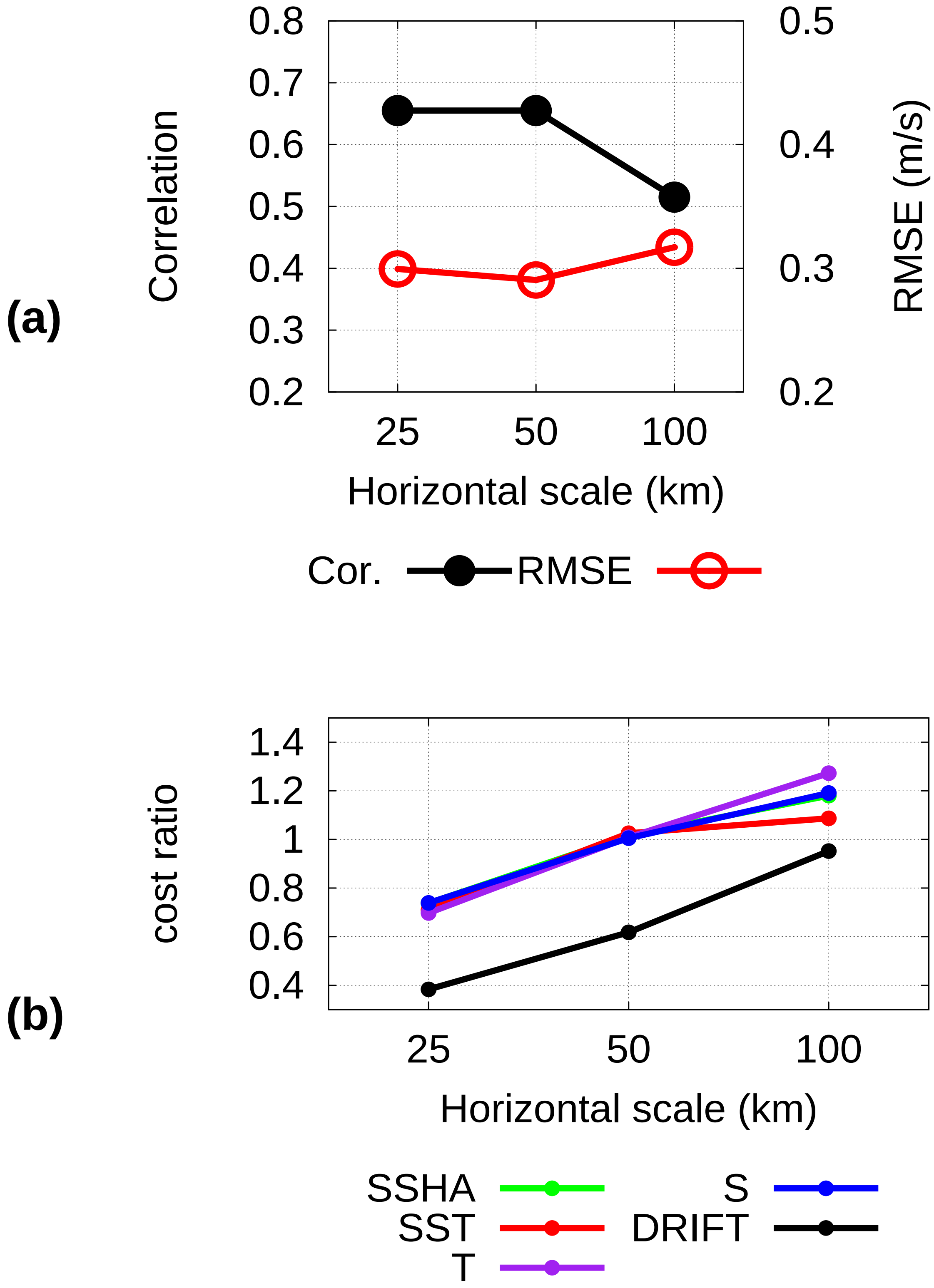

Supplementary Figure 2
